# Marchiafava-Bignami Disease in a Postoperative Non-alcoholic Patient

**DOI:** 10.7759/cureus.71452

**Published:** 2024-10-14

**Authors:** Dhyey Sidhpura, Devagna Mehta, Aastha Nayak, Apara Kothiala

**Affiliations:** 1 Neurology, Gujarat University, Ahmedabad, IND; 2 Medicine, Gujarat Cancer Society (GCS) Medical College, Ahmedabad, IND; 3 Internal Medicine, Smt. Nathiba Hargovandas Lakhmichand (NHL) Municipal Medical College, Ahmedabad, IND; 4 Neurology, Gujarat Cancer Society (GCS) Hospital, Ahmedabad, IND

**Keywords:** marchiafava-bignami disease, mri, non-alcoholic, s:malnutrition, splenium of the corpus callosum, thiamine or vitamin b1 deficiency

## Abstract

Marchiafava-Bignami disease (MBD) is a neuropathological condition characterized by demyelination and necrosis of the corpus callosum. This condition is commonly found in malnourished and alcoholic patients, but it is rarely observed in non-alcoholic individuals. In this case report, we describe a non-alcoholic patient who underwent two consecutive gastrointestinal surgeries. After the surgeries, he developed seizures and was diagnosed with MBD based on a plain head MRI. However, with the proper treatment, his condition stabilized, and he was discharged. This case serves as a beacon of hope, demonstrating that MBD, even in non-alcoholic patients, can be reversible with appropriate imaging and treatment.

## Introduction

A central nervous system disorder characterized by a rare toxic, demyelinating, and necrotic condition of the corpus callosum is known as Marchiafava-Bignami disease (MBD) [1]. It was named after two Italian pathologists, Ettore Marchiafava and Amico Bignami, who initially described it in 1903 to be affiliated with Italian males addicted to crude red wine [2]. They provided examples of individuals with alcohol use disorders who died from convulsions and comas and were found to have necrosis of the corpus callosum during autopsy. Most of the cases related to MBD have been associated with long-term chronic alcoholic and malnutrition patients. Still, it is also seen in some non-alcoholic [2-4].

The role of vitamin B deficiency in MBD cannot be overstated. It has been proposed as the main contributor to this condition. Notably, many patients have shown improvement after being administered vitamin B. However, it's important to note that not all patients respond to this treatment, highlighting the complex nature of MBD. The acute phase of MBD consists of severe neurological manifestations like confusion, dysarthria, seizure, hypertonic limb, and delirium/coma [1].

We report a case of a non-alcoholic male developing MBD-like clinical features and MRI findings after undergoing two consecutive gastrointestinal surgeries.

## Case presentation

We present a comprehensive case of a 37-year-old man who initially sought medical attention for decreased defecation and distention of the abdomen. Upon an X-ray of the abdomen, he was diagnosed with small bowel obstruction and subsequently underwent resection and anastomosis surgery. However, a few days later, he experienced a recurrence of obstruction, leading to a CT scan that revealed postoperative adhesion. This necessitated an exploratory laparotomy and resection and anastomosis with ileostomy. The patient was then kept nil per oral (NPO), IV fluids, and total parental nutrition for 15 days.

**Figure 1:**
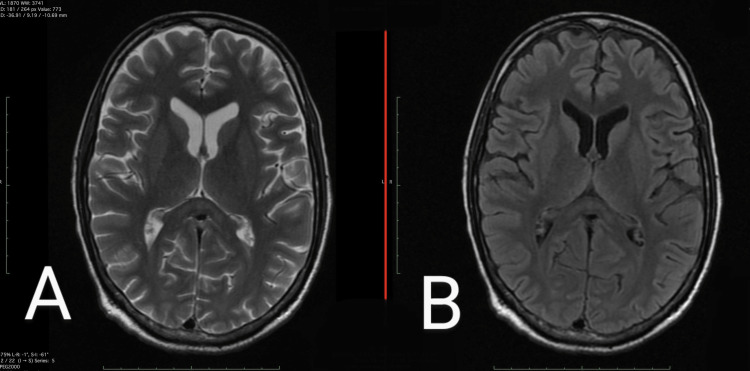
(A) T2W MRI and (B) FLAIR MRI (A) T2W image shows hyperintensity involving the splenium of the corpus callosum. (B) FLAIR image shows subtle hyperintensity involving the splenium of the corpus callosum. FLAIR: fluid-attenuated inversion recovery

**Figure 2:**
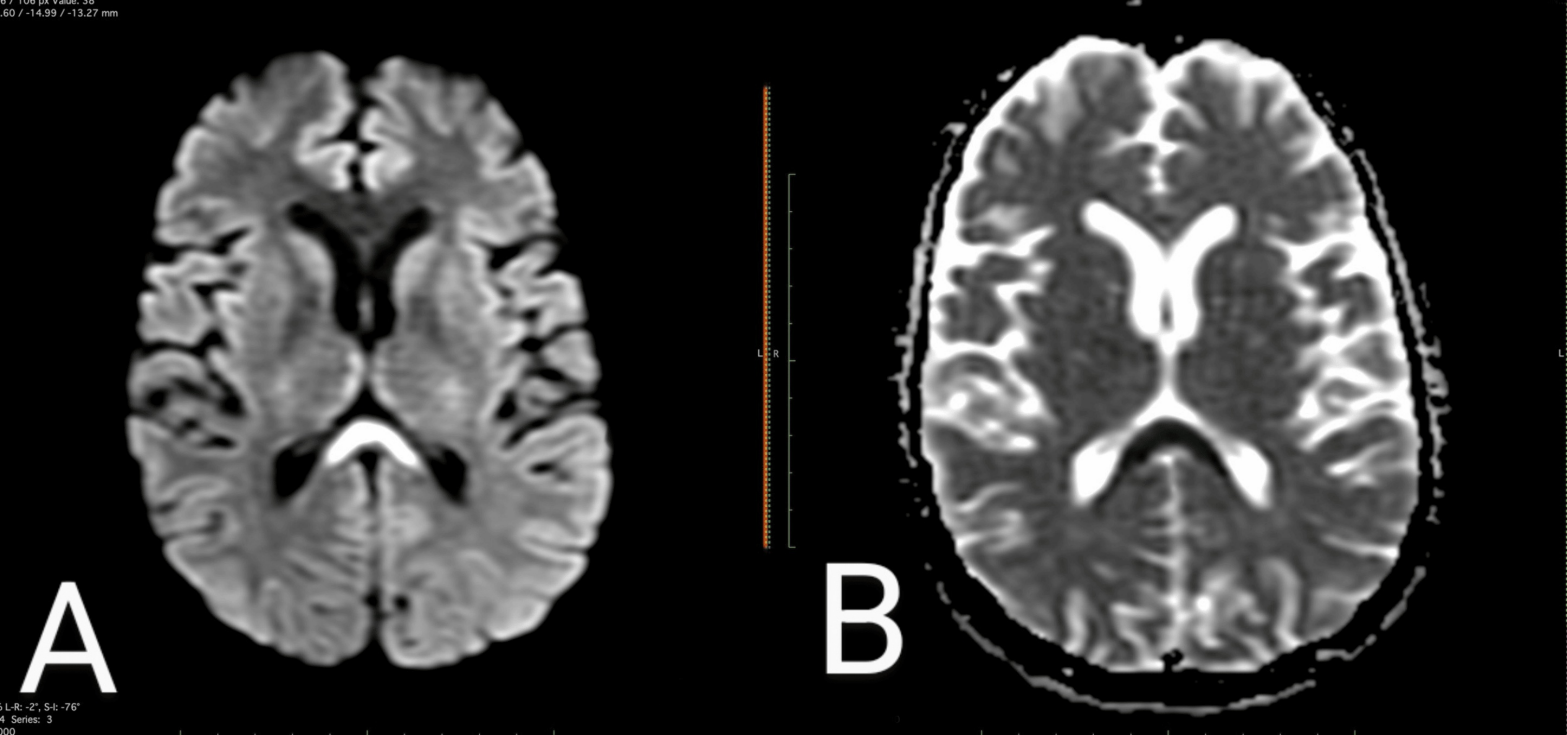
(A) DWI MRI and (B) ADC MRI (A) DWI shows oval hyperintensity throughout the splenium and into the adjacent hemisphere ("boomerang sign"). (B) Corresponding ADC map shows hypointensity due to restricted diffusion involving the splenium of the corpus callosum. DWI: diffusion-weighted imaging; ADC: apparent diffusion coefficient

Once the patient was stable and ready for discharge, he developed a high-grade fever, seizures, and altered mental sensorium. Following this, all the routine investigations were ordered, along with cerebrospinal fluid (CSF) analysis and plain MRI of the brain. On routine investigations, only the white blood cell (WBC) count was elevated at 12000, as seen in Table 1, and CSF analysis was normal, as seen in Table 2. The MRI showed hyperintensity in the splenium of the corpus callosum, suggestive of cytotoxic injury, as seen in Figure 1 and Figure 2.

**Table 1:** Blood investigations Except for the WBC count, all other blood investigations are normal. WBC: white blood cell; BUN: blood urea nitrogen; CRP: C-reactive protein; TSH: thyroid-stimulating hormone; AST: aspartate transferase; ALT: alanine transaminase

Parameters	Patient value	Reference range
WBC count	12	4.5-11 thousand/mL
Blood hemoglobin	10.5	14-17 g/dL
Hematocrit	43	42-50%
Platelets	229	150-450 thousand/mL
S. sodium	137	135-145 mEq/L
S. potassium	3.8	3.5-5 mEq/L
S. bicarbonate	25	22-28 mEq/L
S. chloride	99	95-107 mEq/L
S. BUN	13	5-20 mg/dL
S. creatinine	0.9	0.6-1.2 mg/dL
S. glucose	72	65-100 mg/dL
CRP	0.22	<1.0 mg/L
S. TSH	2.6	0.5-5 μU/mL
S. B12	347	160-100 ng/L
AST	25	8-48 U/L
ALT	32	7-55 U/L
S. bilirubin	0.4	0.3-1 mg/dL

**Table 2:** Normal CSF analysis CSF: cerebrospinal fluid

Characteristics	Values
Pressure	100 mmHg
Color	Clear
Turbidity	Absent
Blood	Absent
Glucose	56 mg/dL
Protein	27 mg/dL
White blood cell counts	3

Based on investigations and clinical presentations, along with the history of major surgeries and parental nutrition, the cytotoxic injury to the corpus callosum was deemed to be due to the deficiency of micronutrients, particularly vitamin B complexes. Thus, the diagnosis of MBD was made.

The patient was treated with injectable thiamine 500 mg IV TDS, a tablet of mecobalamin 1500 mcg daily, and vitamin D3 2000 IU daily for 10 days. After that, the patient started improving and was discharged home in good health.

## Discussion

MBD is characterized by cytotoxic injury to the corpus callosum. Two sets of classifications are present for MBD.

The first classification is the older one during the autopsy era based on the rate of clinical progression; type A was acute/subacute, characterized by coma, seizures, limb hypertonia, and pyramidal tract symptoms, while the rapid onset of dementia, limb hypertonia, dysarthria, and astasia-abasia was considered typical for subacute forms. Progression to death was more lethal in acute/subacute, but later cases of prolonged slow progression gave rise to the type B chronic form.

The second classification is based on the clinical-radiological features of MBD, where type A has diffused corpus callosum involvement with poor prognosis and type B has partial corpus callosum involvement with good reversibility and better prognosis [5].

MBD has many differential diagnoses based on corpus callosum involvement, such as multiple sclerosis, diffuse axonal injury in preceding trauma events, callosal infarction, which is rare due to its rich blood supply, and transient lesion of the splenium. MRI is the most important modality that helps in the early detection and differentiation of MBD from other pathologies [6].

In the etiology of MBD, alcohol is the most common factor associated with MBD. This is believed to be due to toxins in alcohol that can directly affect the corpus callosum. Additionally, severe liver dysfunction in chronic alcoholism can increase ammonia levels, leading to encephalopathy, edema, and demyelination of the corpus callosum. Malnutrition and vitamin deficiency are also commonly associated with MBD, with a significant role attributed to vitamin B complex deficiency, which is frequently related to both chronic alcoholism and malnutrition [7,8]. Other causes are callosal myelinolysis due to sudden fluctuation in serum osmolality [9] and non-alcohol-related conditions like carbon monoxide poisoning, sepsis, cerebral malaria, and sickle cell disease [10,11].

Our case was different in terms of history. The patient did not have alcoholism. However, the patient has a history of two consecutive gastrointestinal operations, both of which were followed by the patient being on NPO and total parenteral nutrition. He developed altered mental status and seizure days following his second surgery, for which he had a plain MRI of the brain that showed characteristic lesions of hyperintensity on the T2 imaging in the splenium of the corpus callosum consistent with the diagnosis of MBD [1]. Differential diagnoses for cytotoxic injury to the corpus callosum were considered during the patient's evaluation.

The patient was treated with injectable thiamine, which significantly improved consciousness and overall health. The rationale for thiamine treatment was based on the fact that thiamine deficiency can negatively impact the ability of cells to control osmotic gradients, potentially leading to cytotoxic edema. One theory suggests that oligodendrocytes, usually found in white matter, may face increased susceptibility to osmotic stress when in close proximity to grey matter [12]. Alternatively, corticosteroids are also used for treatment as they have been shown to decrease inflammatory edema, stabilize the blood-brain barrier, and decrease the production of leucocytes, but evidence suggests that full recovery is achieved by adequate thiamine treatment [13] which is seen in our case report.

## Conclusions

MBD can occur in patients without chronic alcoholism and malnutrition. Patients with a history of extensive surgeries and long-term parenteral nutrition are also prone to developing this condition. This may be due to the decreased nutritional status of the patients. Prompt recognition of the condition by MRI of the brain and administration of proper treatment like thiamine may help reverse acute symptoms developed from inflammatory edema. Further studies are needed to evaluate post-op patients with a similar condition.
